# Towards Detecting Biceps Muscle Fatigue in Gym Activity Using Wearables

**DOI:** 10.3390/s21030759

**Published:** 2021-01-23

**Authors:** Mohamed Elshafei, Emad Shihab

**Affiliations:** Department of Computer Science and Software Engineering, Concordia University, Montreal, QC H3G 1M8, Canada; eshihab@cse.concordia.ca

**Keywords:** human activity recognition, sensors, wearable sensor data, machine learning

## Abstract

Fatigue is a naturally occurring phenomenon during human activities, but it poses a bigger risk for injuries during physically demanding activities, such as gym activities and athletics. Several studies show that bicep muscle fatigue can lead to various injuries that may require up to 22 weeks of treatment. In this work, we adopt a wearable approach to detect biceps muscle fatigue during a bicep concentration curl exercise as an example of a gym activity. Our dataset consists of 3000 bicep curls from twenty middle-aged volunteers at ages between 27 to 30 and Body Mass Index (BMI) ranging between 18 to 28. All volunteers have been gym-goers for at least 1 year with no records of chronic diseases, muscle, or bone surgeries. We encountered two main challenges while collecting our dataset. The first challenge was the dumbbell’s suitability, where we found that a dumbbell weight (4.5 kg) provides the best tradeoff between longer recording sessions and the occurrence of fatigue on exercises. The second challenge is the subjectivity of RPE, where we average the reported RPE with the measured heart rate converted to RPE. We observed from our data that fatigue reduces the biceps’ angular velocity; therefore, it increases the completion time for later sets. We extracted a total of 33 features from our dataset, which have been reduced to 16 features. These features are the most overall representative and correlated with bicep curl movement, yet they are fatigue-specific features. We utilized these features in five machine learning models, which are Generalized Linear Models (GLM), Logistic Regression (LR), Random Forests (RF), Decision Trees (DT), and Feedforward Neural Networks (FNN). We found that using a two-layer FNN achieves an accuracy of 98% and 88% for subject-specific and cross-subject models, respectively. The results presented in this work are useful and represent a solid start for moving into a real-world application for detecting the fatigue level in bicep muscles using wearable sensors as we advise athletes to take fatigue into consideration to avoid fatigue-induced injuries.

## 1. Introduction

Fatigue is a natural outcome of prolonged activities. A previous work [1] classifies fatigue into two types, objective and subjective fatigue. The objective fatigue is generated from performing physical activities, leading to a decrease in the capability to exert mechanical work [2]. In comparison, subjective fatigue is generated from intense mental tasks, leading to a decline in alertness and mental concentration [3]. In this work, we focus on objective fatigue in gym activities, where a disturbance between the production and consumption of metabolic energy occurs due to physically demanding activities. As a result, metabolic waste is accumulated at the cellular level, pushing the body to break its homeostasis state [4]. Previous works [5,6,7] about gym over-training in athletes indicate that fatigue often occurs prior to muscle injuries, where muscles are at their most vulnerable state. These injuries are referred to as fatigue-induced injuries, which can lead to a series of complications, such as substantial loss in muscle strength and flexibility [8]. Thus, our work aims to detect fatigue during gym activity to reduce the risk of fatigue-induced injuries. We chose bicep focus crimping as our research topic because it includes flexing one of the most active skeletal muscles [9,10] in the elbow joint countless times to select, lift, and pull things.

The bicep is a muscle in the anterior compartment of the upper arm, along with the brachialis muscle and the coracobrachialis muscle [11]. It has limited and repetitive movements due to its placement; therefore, these movements stress the bicep structures (e.g., muscle tissues, tendons, or joints) over time, leading to muscle fatigue. Unfortunately, fatigue gradually reduces muscle endurance, until it exceeds the muscle’s stress tolerance, where injuries occur [12]. Commonly, bicep fatigue injuries, such as muscle strain, may require up to 22 weeks of treatment [13,14], while tendon rupture results in a substantial permanent decrease in flexion and extension strength [12]. This may delay athletes’ training schedules or their immediate withdrawal from competitions [15].

### 1.1. Background

Muscle fatigue is a complex and multifaceted phenomenon with various definitions; however, one of the most common definitions of fatigue is “failure to maintain the required force to continue performing a task” [16,17]. Therefore, muscle fatigue usually denotes a transient decrease in the capacity to perform physical actions [18]. While other works [18,19,20,21] quantify fatigue as a decline in the maximal force or power capacity of muscle after performing a task for long periods. Nowadays, several fatigue detection approaches have been proposed in the literature to monitor fatigue and reduce the risk of fatigue-induced injuries. Those approaches are commonly grouped into three main categories [22,23]: the invasive approach, the cardio-respiratory approach and the wearable approach.

#### 1.1.1. The Invasive Approach

The approach is one of the earliest methods used to detect fatigue. This approach usually requires instruments to puncture the skin or contact with the mucosa. A previous work uses blood lactate concentration test to muscle endurance in athletes to reduce the risk of over-training and injuries [24]. The work’s findings show that blood lactate analysis provides high accuracy (up to 97%) in evaluating muscle endurance; however, it often requires several blood samples from the swimmers during and after progressive incremental swimming. Another previous work measures, the lactic acid in the bloodstream to determine the maximal muscle effort that a person can maintain without risking fatigue injuries [25]. The work’s findings indicate that blood lactate concentration levels are significantly different (*P* < 0.05) in muscles during moderate to fatigue intensities. Another previous work measures lactate and creatine kinase levels in the bloodstream to assess the risk of skeletal muscle injuries during a marathon run [26]. The work’s findings indicate that high levels of lactate and creatine kinase can indicate insufficient oxygen intake to the muscles, causing fatigue. In some extreme levels of creatine kinase, the injuries become inevitable. A less painful method was presented in a previous work that measures rectal temperature to predict exercise duration until fatigue occurs in different environmental conditions [27]. The work’s findings show that rectal temperature increased linearly throughout exercise trials and correlated significantly (r = 0.92) with exerted force to predict the duration of exercise to fatigue. Although the aforementioned approach provides an accurate estimation of fatigue, a practical drawback is that it requires several blood samples during different incremental exercise stages.

#### 1.1.2. The Cardio-Respiratory Approach

The approach is based on a person’s metabolic system and it requires a face mask to measure the rate of oxygen intake during an exercise. Some studies refer to this approach as VO${}_{2}$ max, which stands for the maximum volume of oxygen consumption measured during incremental exercise. A previous work measures the circulatory and respiratory systems’ ability to supply oxygen (O${}_{2}$) to skeletal muscles during sustained physical activity without risking fatigue injuries [28]. The work’s findings indicate that Oxygen consumption provides an accurate indication (>94%) of exercise intensity, yet this requires costly equipment and technical expertise, which outweighs its usefulness for quantifying load during routine training. Another previous work measures the volume of oxygen consumption (VO${}_{2}$) to determine the time to reach fatigue between various runners [29]. The work’s findings indicate that the runner’s body requires far more oxygen consumption at the maximum speed, which cannot be satisfied; therefore, the oxygen debt continuously increases, causing the body to slow down—also known as fatigue. Another previous work measures the volume of oxygen consumption (VO${}_{2}$) to study the development of muscle fatigue in healthy humans during incremental cycling [26]. The work’s findings indicate that the rate of oxygen consumption increases as resistance increases to postpone the development of muscle fatigue evoked by incremental cycling. Another previous work shows that a reduction in work efficiency, also known as fatigue, results from an additional energy cost and oxygen requirement during high-intensity exercise [30]. The work’s findings show that muscle fatigue can be detected while performing the VO${}_{2}$ max test, as the body cannot maintain maximum VO${}_{2}$ values until it fully recovers from fatigue. Although the aforementioned approach provides an accurate estimation of fatigue, a practical drawback is that the required setup of equipment is too complex to be operated singularly.

#### 1.1.3. The Wearable Approach

The approach is a promising approach which recently developed to overcome such previous drawbacks. A previous work [31] uses mouth-guard and Galvanic Skin Response (GSR) biosensors to monitor athletes’ metabolites from saliva and eccrine sweat continuously. The work’s findings show that monitoring biomarkers from saliva or sweat allows us to detect up to 95% of over-training during incremental training accurately. Another previous work uses an Inertial Measurement Unit (IMU) to collect data from outdoor marathon runners and analyzes the data using Machine Learning to predict fatigue [32]. Another previous work used wearable Electromyography (EMG) to evaluate workers’ muscle fatigue as a means of assessing their physical stress on construction sites [33].

### 1.2. Our Contributions

In this work, we select concentration bicep curls as an example of a gym activity. We believe such an activity is a good choice to detect bicep muscle fatigue because it focuses on the muscle’s repetitive movements. We started by collecting our dataset for concentration biceps curls from 20 volunteers aged between 20–35 using a wearable IMU placed on the wrist. Additionally, we recorded the heart rate of the volunteers during the exercise using Apple Watch Series 4. Then, we generated 33 features, which were reduced to the 16 most significant features. After that, we trained five Machine Learning models to detect bicep muscle fatigue during the exercise to be subject-specific and across-subjects. In this work, we answer three Research Questions (RQs). In RQ1, we search for the most significant features to detect bicep muscle fatigue. Gym activities and athletics have a repetitive nature, where participants have to train their muscles through incremental exercises. Therefore, the collected data from these activities inherit repetitive patterns. However, these patterns may not last for long in the presence of fatigue. Therefore, we have to find the changes in data patterns and extract the most significant features from these changes to detect fatigue once it kicks in. In RQ2, we measure the performance of subject-specific models in detecting biceps muscle fatigue. These models are parameterized to fit separate individuals with distinct fatigue patterns. Although these models perform highly, they require previous data from each individual. In comparison, RQ3 aims to measure the performance of cross-subject models, which are parameterized to fit a group of individuals. These models can utilize data from previous individuals to fit new users. Our contributions are summarized as follows:

1. Detecting bicep muscle fatigue during exercise to prevent fatigue-induced injuries and, as a result, avoid its economic impacts [34,35].
2. Providing a more practical and feasible approach in daily life compared to early approaches [36,37,38].
3. Highlighting a number of challenges and solutions related to data collection and processing
4. Publishing a biceps muscle fatigue dataset in the form of a concentration curl exercise (bicep fatigue dataset—https://zenodo.org/record/3698242#.XmFZ5qhKguU).

The rest of the paper is structured as follows, Section 2 demonstrates how we collected our dataset, labeled its entries, and addressed the related challenges. Section 3 illustrates our approach to data processing, feature extraction, and experimental setup. In Section 4 we answer the three research questions about detecting biceps muscle fatigue using wearables. In Section 5, we discuss RPE risk, compare our approach to previous approaches, interpret the findings and fatigue implications. Section 6 concludes our paper.

## 2. Materials: Data Collection and Related Challenges

This section looks into the details of our dataset collection process and discusses the related challenges. Additionally, it illustrates the possible solutions to overcome the challenges to provide a high-quality dataset for our study.

### 2.1. Collecting Data

Our biceps muscle fatigue dataset must contain a sufficient number of concentration bicep curls for three purposes: (1) to have enough data entries that capture bicep muscle fatigue; (2) to observe the variations of fatigue across the volunteers during the exercise; (3) to capture the kinetic changes that occur due to fatigue during the exercise. Therefore, we asked twenty volunteers aged between 29–33 to perform concentration curls with a dumbbell. We selected middle-aged volunteers because athletes usually notice physical declines at ages between 27 to 30 [39,40]. The twenty volunteers were diverse in Body Mass Index (BMI) [41], ranging between 18–28 BMI, to include underweight, normal weight, and overweight. Additionally, all of the volunteers had been gym-goers for at least 1 year. None of the volunteers had chronic diseases or had been through muscle or bone surgeries. None of the volunteers were taking drugs or substances that were expected to affect their physical performance.

To help us recognize the presence of fatigue, we explained the Borg’s scale to each volunteer. The Borg’s scale is used for evaluating the rate of perceived exertion (RPE), which is a subjective measurement of fatigue intensity within sport science. RPE has been proven to model a person’s performance better in the real-world compared to only heart rate monitoring [42]; thus, RPE is an appropriate and validated marker of a volunteer’s fatigue [43]. Table 1 shows the Borg’s scale explained to the volunteers to allow them to rate their level of exertion during the exercise. At scales 6–10, the volunteer reports no to light feelings of exertion. At the scale 11–14, the volunteer reports fairly light to moderate levels of exertion. At the scale 15 or higher, the volunteer reports vigorous level of exertion. Borg ratings range from 6 to 20, whereby multiplying the Borg rating by ten, we can estimate the person’s heart rate during the activity. This serves as a way to strengthen the validity of reported RPE for each volunteer. For example, if a volunteer reports 15 on the Borg rating, we multiply 15 by ten to get the volunteer’s estimated heart rate, which should be close to the measured heart rate by the Apple Watch.

**Figure 1:**
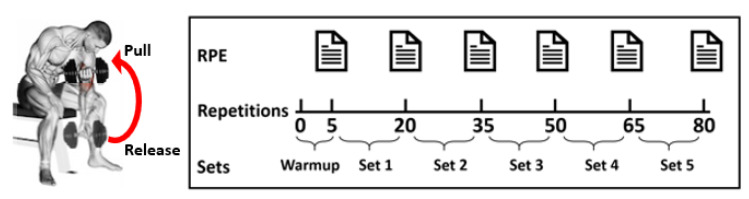
Visualization of data acquisition sessions of concentration bicep curl exercise.

We start our data session by placing a 50 Hz IMU on the volunteer’s wrist to measure linear and angular velocities through a 3-axes accelerometer, gyroscope, and magnetometer for a total of nine data signals. To measure the volunteer’s heart rate, we place the Apple Watch Series 4 on the opposite wrist. The watch measures an average number of heartbeats per minute, with the capability of measuring up to 210 beats per minute. As mentioned, this is used to strengthen the validity of reported RPE for each volunteer. Figure 1 illustrates a data collection session for a concentration bicep curl exercise where each volunteer’s data collection session starts with five repetitions to warm-up, followed by 15 repetitions per set for a total of five sets. The volunteer reports his/her RPE values for each set, including the warm-up, yielding six RPE values. Then, we ask the volunteer to repeat the exercise using the other arm. Additionally, we explain the exercise to each volunteer as the following:

1. We ask the volunteer to sit down on a flat bench with bent knees and one dumbbell between the volunteer’s legs.
2. We ask the volunteer to pick up the dumbbell with the right hand while placing the right elbow on the top of the inner right thigh. This is the release position.
3. We ask the volunteer to pull up the dumbbell by only moving the forearms and contracting the biceps while breathing out. Once the biceps are fully contracted, and the dumbbells are at shoulder level, we ask the volunteer to hold the pull position for a second to guarantee a good contraction.
4. Finally, we ask the volunteer to slowly bring the dumbbells back to the release position while breathing in. Then, we ask the volunteer to repeat the exercise for 15 repetitions; after that, repeating the exercise with the left arm.

To summarize, our dataset consists of 3000 concentration bicep curl repetitions from twenty volunteers, where each repetition required approximately 2 s to complete. Given the fact that we used a 50 Hz IMU unit, a single repetition is captured in approximately 100 data samples. As a result, this allows us to distinctly capture signs of fatigue as muscle performance and exerted force decline overtime.

### 2.2. Data Collection Challenges

We encounter three major challenges during our data collection procedure: (1) dumbbell suitability, (2) RPE’s subjectivity, and (3) RPE’s familiarity. We believe that selecting a certain dumbbell weight for collecting data from different volunteers may cause volunteers to get exhausted quickly, hindering us from capturing fatigue over time. This could cause a steady decline in muscle performance and its exerted force to plummet, which is less likely to occur naturally. Using subjective measures, such as RPE, might introduce a dependency between the correctness of selected Borg rating and volunteers’ awareness. Additionally, introducing the RPE to a volunteer for the first time may cause him/her to misevaluate his/her perceived exertion rate.

The first challenge we encountered is the suitability of the dumbbell. The process of collecting data from 20 volunteers raises the issue that not all volunteers can equally perform the exercise. To address this challenge, we provided each volunteer four dumbbells: (1) light-weight includes 1.1 kg and 2.2 kg dumbbells, (2) medium-weight includes a 4.5 kg dumbbell, and (3) heavy-weight includes a 9 kg dumbbell. Then, we asked each volunteer to use them to perform at least 2 sets of bicep concentration curl repetitions until they start feeling fatigued. We observe that using light-weight dumbbells provides us with a high number of repetitions, 42–31 repetitions, but a low number of 3–4 sets over a long time of approximately 2 min ± 25 s per set. This leads to a long recording session with a lot of similar data entries until volunteers reach fatigue. On the other hand, a heavy-weight dumbbell provides us with a lower number of repetitions—9 repetitions, and a low number of 3 sets—but in a short time of approximately 1 min ± 10 s per set. This leads to shorter recording sessions with fewer data entries, which do not capture kinetic changes clearly throughout the exercise because volunteers reach fatigue quickly. The medium-weight dumbbell provides us with a moderate number of repetitions, 16 repetitions, but higher numbers of five sets with a slightly longer time of approximately 1 min ± 17 s per set. This allows us to maintain a short recording session with a slightly higher number of data entries; moreover, we can distinctly capture the kinetic changes throughout the exercise as the volunteers gradually reach fatigue.

The second challenge we encountered is the subjectivity of RPE, where volunteers may rate their exertion level differently based on their feeling of exhaustion. This issue may lead them to inaccurately report their levels of exertion throughout the exercise. In the rare cases of dissimilarity between the Borg scale and the measured heart rates, we average the reported RPE with the measured heart rate converted to RPE, as similarly done in previous work [44]. The third challenge we encountered is RPE’s familiarity. In the beginning, volunteers were unfamiliar with the RPE before the data collection sessions. This may lead them to rate their exertion level incorrectly, which worsens the effect of the previous challenge if it is not appropriately addressed. To address both of these challenges, we collected the data over several sessions to help volunteers gain experience with the scale as they performed more sets. Then, we applied min–max normalization to the RPE value, based on the current set to account for subjective differences in RPE. We fixed the maximum value at 20, which is the highest RPE on the Borg scale, to avoid the current label set, depending on the future set. However, we set the minimum value, based on the RPE reported after the warm-up, ranging from 10 to 12.

## 3. Methods: Data Processing and Experiment Setup

This section explains our approach to fatigue detection by defining bicep fatigue, data processing, and feature extraction. Then, we describe how we set up our experiment.

### 3.1. Data Processing and Feature Extraction

**Figure 2:**
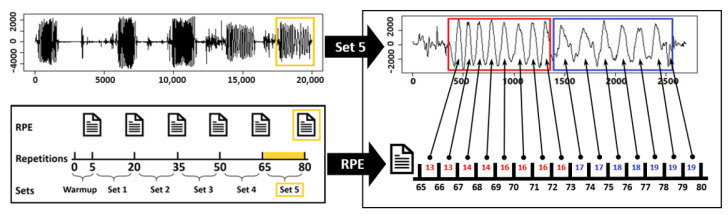
Extracting and labeling bicep concentration curl repetitions from the fifth set.

We started processing the data collected from each volunteer by extracting their five sets of concentration curls. Then, for each set, we associated each concentration curl with the RPE values reported by the volunteers to identify whether a repetition contains fatigue or not. For illustration, Figure 2 shows how we extracted and labeled repetitions in the fifth set. The troughs indicate that the volunteer reached the release position while peaks indicate that he/she reached the contraction position, as demonstrated in Section 2.1. We extracted and labeled each repetition manually, according to the RPE values reported for the set. For example, Figure 2 shows that a group of eight non-fatigue and seven fatigue repetitions were extracted from the fifth set. Additionally, we observed that a non-fatigue repetition was relatively symmetrical; on the other hand, the fatigue repetition had a relatively positive skew. We repeated the same process for the remaining sets until we finished all twenty of the volunteers data. In total, we were able to extract and label a total of 3000 repetitions recorded from 9 time-series signals with one IMU. These signals are the three-dimensional Cartesian coordinates (x,y,z) for gyroscope, magnetometer, and accelerometer. Additionally, we increased the collected data by computing two additional signals from the accelerometer data from an IMU, as the following:

- Total Acceleration: this is the vector sum of the tangential and centripetal accelerations, which makes it a place-independent signal, which means it does not rely on the exact attachment of the accelerometer because it combines *x*, *y*, and *z* acceleration signals at time $t_{i}$ to compute a total acceleration, defined as: $\sqrt{a_{x_{i}}^{2}+a_{y_{i}}^{2}+a_{z_{i}}^{2}}$.
- Exerted Force: $F_{exerted}=m\times a$ is the exerted force by the a volunteer to lift the dumble. $F_{exerted}$ is calculated by multiplying the mass *m* of the lifted dumble by acceleration *a*.

**Figure 3:**
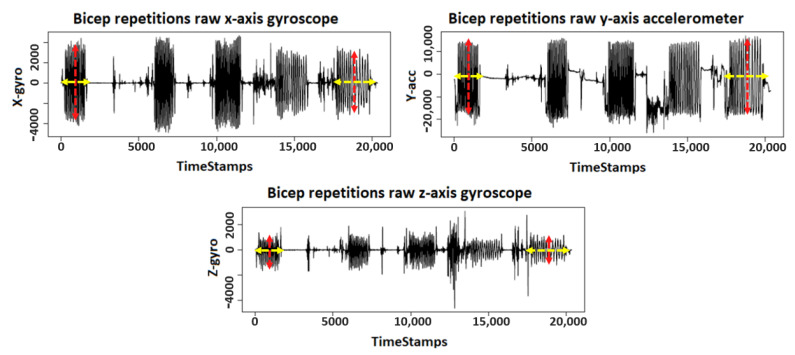
A visualization of the changes that occurred to muscle endurance and angular velocity due to fatigue presence.

In this work, we used wearables to measure the decline in bicep muscle performance through changes in the kinetic and the exerted force of the muscle during the exercise and associated such changes to the reported RPE. Based on these change patterns, we can define bicep fatigue; hence, we extracted a set of suitable features to detect fatigue. The axes reported in Table 2 and Table 3 represent the following: (1) the X-axis represents vertical displacement, which is the distance between the highest and lowest positions of the volunteer’s hand during bicep extension and flexion; (2) the Y-axis represents horizontal displacement, which is the sideways vibration of the volunteer’s hand during bicep extension and flexion; (3) the Z-axis represents depth displacement, which represent the farthest and nearest positions of the volunteer’s hand from their body during bicep extension and flexion. Figure 3 shows the data pattern changes along the horizontal axis, which indicates the changes in completion time, whereas the vertical axis indicates the changes in angular velocity according to muscular endurance [45]. As time passed by, we observed the loss in muscle endurance and angular velocity during fatigue presence using the gyroscope and the accelerometer. We selected the X-axis from the gyroscope and Y-axis from the accelerometer because they provided the best visualization for the angular velocity and sideways vibration of the volunteer’s hand. On the other hand, we selected the Z-axis from the gyroscope, because it provides the best visualization of the farthest and nearest positions of the volunteer’s hand. Table 2 shows the increase in completion time for each set, relative to the first set. Table 3 shows the changes in muscular endurance for each of the five sets as the fatigue accumulates during repetitions in the later sets. As time passed, we observed that later sets required a longer time for completion because of the decline in angular velocity. This is because, the later the sets are, the more fatigue repetitions they contain. In fact, this is a similar observation to previous studies [46,47] that measure the effort during resistance exercises by using velocity loss as a variable. In our case, we used the loss in angular velocity from the gyroscope. Therefore, we observed that fatigue decreased muscular endurance, according to the X and Z axes from the gyroscope by an average of –2.4% and –3.9%, respectively.

After we extracted all repetitions individually, we computed 33 features by computing the mean, mean absolute deviation (AAD), and standard deviation (SD) for all twelve signals. Next, we needed to evaluate these features to answer the research questions in Section 4.

### 3.2. Experiment Setup

Our main goal in this section is to assess the reliability of the extracted features in detecting fatigue during concentration curls exercise. In addition, we evaluate how accurately we can detect bicep muscle fatigue subject-specifically and across-subjects. In this work, we used five Machine Learning/classification models [48] to detect bicep muscle fatigue in concentration curl repetitions. The first model was the Generalized Linear Model (GLM), which was adopted to analyze and count the number of walking steps in a previous study [49]. The second model was the Logistic Regression (LR) model, which was used to analyze and detect human activities in a previous study [50]. The third model is the Random Forest (RF) model, which was used to detect and classify human actions using wearable motion sensor networks in a previous study [51]. The fourth model is the Decision Trees (DT) model, which was used to count and classify ambulatory activities using eight plantar pressure sensors within smart shoes in a previous study [52]. The fifth model is Feedforward Neural Networks (FNN), which was used to detect and count repetitions for complex physical exercises in a previous study [53]. Furthermore, we took into consideration two approaches for each model—the subject-specific and cross-subject approaches:

- Subject-specific model: this is a personalized model for each participant and it uses only the data of that participant. This model would work well if each subject had a unique pattern in response to fatigue accumulation.
- Cross-subject model: this is a single model for all participants. It leverages data from all participants following the assumption that multiple subjects will have similar changes in style as a response to fatigue.

A cross-subject model is optimized for working with a large number of users, which is more realistic in real-world applications. On the other hand, a subject-specific model is tailored to individual data, tending to outperform the cross-subject model.

#### 3.2.1. RQ1: What Are the Most Significant Features to Detect Bicep Muscles Fatigue?

We believe that the performance of some models can degrade when including input features that are not relevant to the target output. The presence of such non-informative features can add uncertainty to the detection and reduce the overall effectiveness of the model. Previous works [54,55] show that some features can be highly correlated with others, where it adds redundant or no valuable information to the model, inducing unnecessary noise that may hinder the model’s performance.

To address this research question, we extracted all the fatigue repetitions into a subset, so that we have a complete dataset and a fatigue subset. Then, we extracted two sets of the 33 features, a set from the complete dataset and another set from the fatigue subset. Each set of features includes the mean, AAD, and SD of exerted force, total acceleration, 3-axes of gyroscope, 3-axes of magnetometer, and 3-axes of accelerometer. After that, we applied a filter-based feature selection using Spearman’s rank with a significance allowance of 0.1 on the features extracted from the fatigue subset. This helps us to identify the most correlated features with the fatigue reported RPE values. Then, we applied a filter-based feature selection using Spearman’s rank with a significance allowance of 0.1 on the features extracted from the complete dataset. This helps us to identify the most correlated features with the overall reported RPE values. After that, we compared the two sets of extracted features to select the overlapping features, which are, overall, the most correlated with the reported RPE, yet are fatigue specific features. Next, we trained Machine Learning models to detect bicep muscle fatigue repetitions during the exercise of concentration biceps curls. This step aims to evaluate how accurately we can detect bicep muscle fatigue subject-specifically and across-subjects.

#### 3.2.2. RQ2: How Accurately Can We Detect Biceps Muscles Fatigue Using Subject-Specific Model?

Thus far, we were able to reduce extracted features from 33 to 16 features. Now, we have to ensure that our approach can achieve high performance; therefore, we want to examine the accuracy, precision, recall, and F1 for all predictive models built on these features. For simplicity purposes, we start with subject-specificity, where we assess the reliability of our work and its ability to predict fatigue, for a specific subject, across different periods of time.

(1) $$Accuracy=\frac{True(Fatigue+NonFatigue)}{True(Fatigue+NonFatigue)+False(Fatigue+NonFatigue)}$$

(2) $$Precision=\frac{True(Fatigue)}{True(Fatigue)+False(Fatigue)}$$

(3) $$Recall=\frac{True(Fatigue)}{True(Fatigue)+False(NonFatigue)}$$

(4) $$F1=\frac{2*Precision*Recall}{Precision+Recall}$$

To address this research question, we utilized the 16 most significant features to build five detection models. We used these models to predict the Borg rating for each repetition. We calculated the accuracy using the confusion matrix shown in Table 4, where non-fatigue repetition represents a Borg score from 6 to 16, and fatigue status represents a Borg score from 17 to 20. We calculated the accuracy using Equation (1), precision using Equation (2), recall using Equation (3), and F1 using Equation (4).

#### 3.2.3. RQ3: Can We Build an Accurate Cross-Subject Model to Detect Biceps Muscles Fatigue?

So far, we were able to build and examine five subject-specific models using the 16 most significant features. Now, we have to assess the generality of the extracted features and the ability to detect fatigue across different subjects; therefore, we want to examine the accuracy, precision, recall, and F1 for all predictive models built on the same features but tested on a different subject.

**Figure 4:**
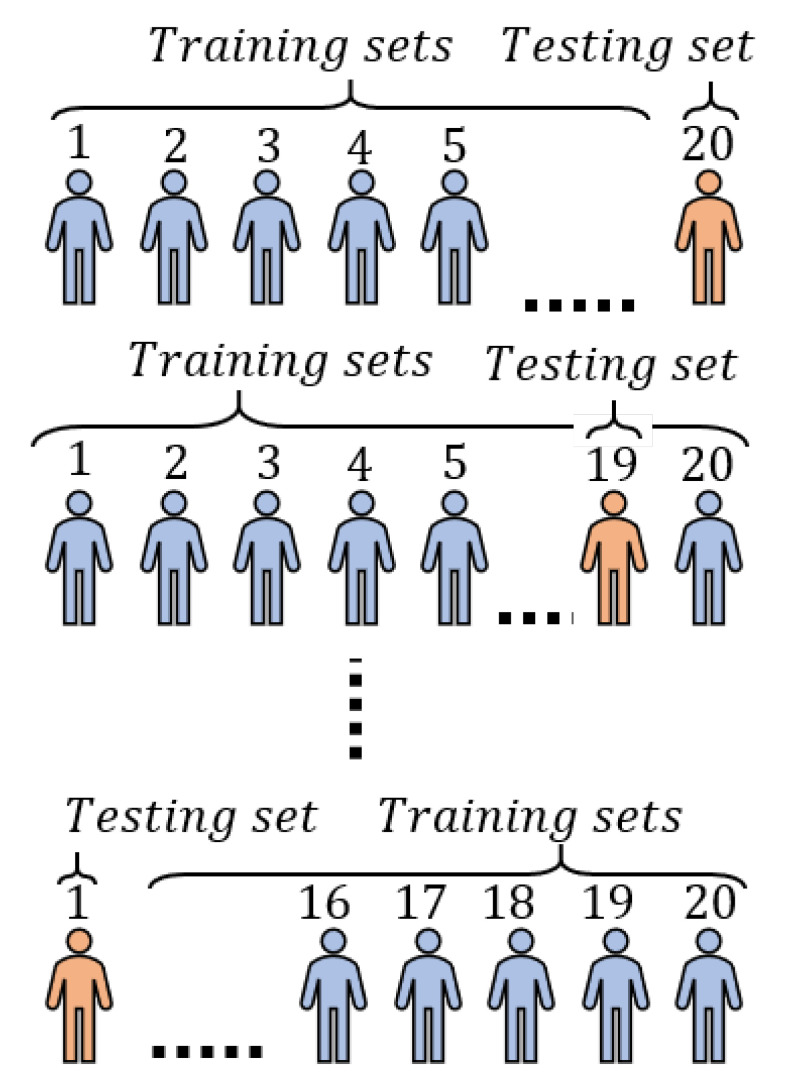
A representation of leave-one-out cross validation for a model using the 20 volunteers’ datasets.

We utilized the 16 extracted features with the five models, however, we used leave-one-out cross validation (LOOCV). This is the same as a K-fold cross-validation, with K = 20 being equal to the number of volunteers. Figure 4 shows that, for a model, in the first iteration, we used 19 volunteers’ datasets for training, excluding the 20th volunteer’s dataset, which we used for testing the model to predict the Borg rating for his bicep repetitions. Then, we calculated the precision, recall, and accuracy. Similarly, in the second iteration, we uses 19 volunteers’ dataset for training, excluding the 19th volunteer’s dataset, which we used for testing the model and calculating the precision, recall, and accuracy. In the 20th iteration, we should have used all volunteers’ datasets for testing, except for the first volunteer’s dataset; therefore, we train the model using all the 19 volunteers’ datasets, then used the first volunteer’s dataset for testing the model and calculating the precision, recall, and accuracy. Finally, we computed the average values for precision, recall, and accuracy for the model using the same equations and the confusion matrix presented in RQ2. LOOCV is usually very expensive from a computational point of view, because of the large number of times that the training process is repeated. This means that we extracted the 16 most significant features from all subjects, then trained each model using the features extracted, excluding one subject. As a result, we were able to execute 20 iterations of training and testing for each model using each set of subject data as a testing set individually.

## 4. Results

In this section, we present the answers for the three asked research questions in Section 3.2 and the conclusions drawn.

### 4.1. RQ1 Results: What Are the Most Significant Features to Detect Bicep Muscles Fatigue?

**Figure 5:**
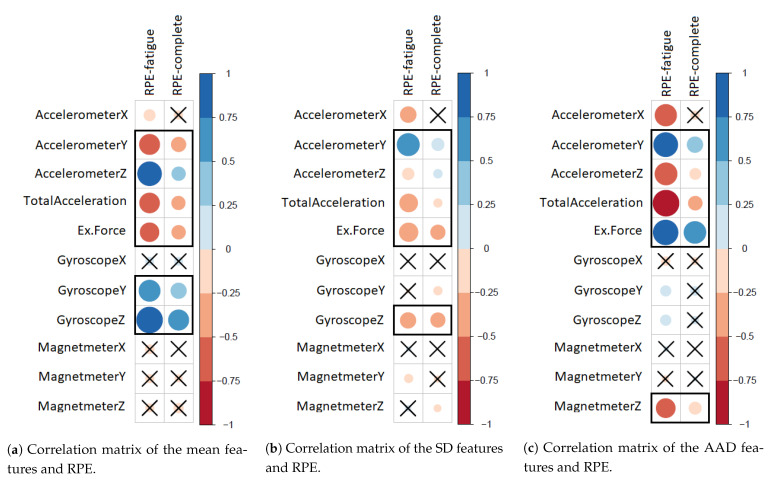
Graphical display of the differences in the correlation matrices of the 33 extracted features (mean, SD, AAD) and the RPE values in the fatigue subset and our complete dataset.

Figure 5 presents the correlation matrices for 33 extracted features and the RPE values in the fatigue subset and our complete dataset. The positive and negative correlations are displayed in blue and red color, respectively. Additionally, the color intensity and the size of the circle are proportional to the correlation coefficients, whereas the insignificant correlations are marked with ×. Figure 5a shows that the reported RPE values have overlapping significant correlations with 6 out of 11 mean features extracted from the fatigue subset and our complete dataset. These six significant features are (Y,Z)-Accelerometer, (Y,Z)-Gyroscope, total acceleration, and exerted force. In addition, Figure 5b shows that the reported RPE values have overlapping significant correlations with 5 out of 11 SD features extracted from the fatigue subset and our complete dataset. These five significant features are (Y,Z)-Accelerometer, (Z)-Gyroscope, total acceleration, and exerted force. Furthermore, Figure 5c shows that the reported RPE values have overlapping significant correlations with 5 out of 11 AAD features extracted from the fatigue subset and our complete dataset. These five significant features are (Y,Z)-Accelerometer, (Z)-Magnetmeter, total acceleration, and exerted force. We can observe that the overlapped features remain significant and have higher correlation coefficient values to fatigue-reported RPE values, which indicate that these features are valuable to detect bicep muscle fatigue.

RQ1 Conclusion: We extracted the 16 overlapping features highlighted in Table 5, which are, overall, the most correlated with the reported RPE, yet fatigue specific features. We came up with these feature after applying spearman’s rank correlation coefficient to evaluate the correlation between 33 extracted features and the reported RPE in the fatigue subset and our complete dataset.

### 4.2. RQ2 Results: How Accurately Can We Predict Subject-Specific Biceps Fatigue?

In Table 6, we present the average subject-specific validations results using the five models. Two-layer Feedforward Neural Networks seem to outperform all other models in terms of precision (95%), recall (93%), accuracy (94%), and F1-measure (94%). We did expect such high performance from FNN, compared to other models, as this occurred in previous studies [56,57,58,59]. These studies show that neural networks have significantly better pattern recognition, compared to other machine learning models; especially, when it comes to periodic activities where extracted features inherit periodicity. Moreover, a popular reason for FNN performance supremacy is that it is robust against small-to-moderate changes in the data which if compared to DT this can cause a large change in the structure of the tree causing instability. On the other hand, we can already observe such effect on DT, which seems to have the lowest performance among the five models in terms of precision (66%), recall (61%), accuracy (58%), and F1-measure (63%). Although we addressed the changes in data in Section 3.2, in which their resonance was amplified if it existed in periodic data which were used to train DT. Meanwhile, the Generalized Linear Model maintained the averaged performance across the five models in terms of precision (86%), recall (83%), accuracy (84%), and F1-measure (84%).

RQ2 Conclusion: Our findings show that our approach achieved high performance for subject-specificity in terms of precision (95%), recall (93%), accuracy (94%), and F1-measure (94%), using the two-layer Feedforward Neural Network. Additionally, our findings complement other studies, as we observed that FNN outperforms all other models, as previously presented [56,57,58,59].

### 4.3. RQ3 Results: Can We Build an Accurate Cross-Subject Fatigue Detection Model?

In Table 7, we present the average cross-subject validation results using the five predictive models. Two-layers Feedforward Neural Networks seems to outperforms all the other models in terms of precision (87%), recall (89%), accuracy (88%), and F1-measure (88%). In the case of cross-subject, we did expect FNN to maintain a superior performance compared to other models as this occurred in a previous study on detecting fatigue while driving where it did archive 95.8% of cross-validation accuracy [60]. Reconsidering the reasons mentioned in RQ2, the cross-subject test amplifies the effect of small-to-moderate changes in the data, causing instability in the structure of DT. Therefore, we can already observe such an effect on DT, which seems to significantly drop DT performance in terms of precision (47%), recall (49%), accuracy (43%), and F1-measure (48%). Meanwhile, the Generalized Linear Model maintains the average performance across the five models in terms of precision (78%), recall (71%), accuracy (75%), and F1-measure (74%).

RQ3 Conclusion: Our findings show that our approach achieves high performance for cross-specific in terms of precision (87%), recall (89%), accuracy (88%), and F1-measure (88%) using the two-layer Feedforward Neural Network. Additionally, our findings complement another study, as we observed that FNN maintains its superior performance in cross-subject validation, as previously presented [60].

## 5. Discussion

Although RPE may pose a risk due to subjectivity, individual differences, and physical fitness level, we were careful to select volunteers closer to athletes. They are middle-aged volunteers because athletes usually notice physical declines between 27 to 30 years [39,40] and BMI [41] ranging between 18 and 28. Additionally, they have been gym-goers for at least 1 year. However, we constructed a gold standard to counter RPE risks by combining heart rate and RPE value. The Borg rating ranges from 6 to 20, whereby multiplying the Borg rating by ten, we can estimate the person’s heart rate during the activity. This serves as a way to strengthen the validity of the reported RPE for each volunteer. We employed a tolerance of ±10 bpm to convert the measured heart rate to RPE before verifying the convergence between the Borg scale and the measured heart rate. We only found a small minority of repetitions where the heart rate metrics and the Borg scale diverge. In the worst case scenario, we found a volunteer that reported 5 out of 80 repetitions (6.2%) with a Borg scale dissimilar to the measured heart rate. To address these cases, we averaged between the measured heart rate converted to RPE and the reported RPE, as done in similar work [44]. For example, if a volunteer reported a repetition of an RPE of 17, but we measured their heart rate as 145 bpm, we first converted the heart rate to RPE: 14.5. Then we averaged both metrics, (17+14.5)/2=15.75, rounded up to 16. The RPE of 16 is used for the labeling of this repetition (repetition without fatigue).

Our work adopts the wearable approach to detect bicep muscle fatigue using a wearable IMU and smartwatch. This allows us to overcome drawbacks from early approaches, such as complexity, discomfort, and invasion. First, regarding complexity, our work is simple compared to the works of early approaches. Our work requires only an IMU and a smartwatch as data acquisition devices, which are fairly easy to interact with and setup, whereas other approaches may require expert supervision, such as fatigue monitoring systems [61]. Second, regarding discomfort, our work spins around portability and being light-weight compared to the works of early approaches. Our work used a Neblina IMU and an Apple Watch Series 4 that weigh 1.3 g and 40 g, respectively. Such light-weight devices do not hinder or interfere with the person’s activity, whereas other approaches may require a face mask to measure oxygen consumption VO${}_{2}$, which is inconvenient in public and often hinders a person’s comfort [29]. Third, regarding invasion, our work was non-invasive compared to the works of early approaches. Our work id not introduce any instruments into a person’s body or require a puncture of the skin. We simply attached the Neblina IMU and Apple Watch Series 4 on the person’s wrist; the invasive approaches always require blood lactate [25], creatine kinase [26], or rectal temperature [27].

In this work, we present 16 overlapping features highlighted in Table 5, which are the most fatigue-specific and highly correlated with the reported RPE. However, if we look at the Table from another perspective, we would notice six non-overlapping but significant features under the fatigue subset. This means fatigue may disturb data patterns over time, alternating features from significant into insignificant status or vice versa. Previous work [62] suggests a decrease in the mean power frequency of the accelerometer readings trend with increasing bicep muscle fatigue, altering some of the extracted features from significant to insignificant. A fatigue implication can be viewed if we consider building a model to detect/count repetitions of biceps concentration curls and neglect fatigue’s significant features. This may cause the model failure to in detecting/counting the fatigued biceps concentration curls as repetitions. The reason for such a dilemma is fatigue affects the collected data; its impacts will extend to the extracted features from the same data. A previous work [63] shows that muscle fatigue affects the electromyography (EMG) data signals collected from biceps by increasing the Root Mean Square Error (RMSEs), leading to the misclassification of some activities.

## 6. Conclusions

This paper presents bicep fatigue as an interesting problem that may lead to fatigue-induced injuries during physically demanding activities. We started with a dataset collected from twenty volunteers with ages and BMI ranging between 29–33 and 18–28, respectively. During our data collection, we encountered two main challenges. The first challenge is the dumbbell’s suitability, where we found that a dumbbell weight of 4.5 kg provides the best tradeoff between longer recording sessions and the occurrence of fatigue on exercises. The second challenge is the subjectivity of RPE, where we average the reported RPE with the measured heart rate converted to RPE. The third challenge is RPE’s familiarity; therefore, we collected the data over several sessions to help volunteers gain experience with the scale as they performed more sets. In the end, our dataset consisted of 3000 concentration bicep curl repetitions from twenty participants, which we used to extract 33 features. We reduced the features to the 16 most representative, then utilized these features in five machine learning models. We found that using a two-layer FNN achieved an accuracy of 98% and 88% for subject-specific and cross-subject models, respectively. Moreover, our methodology aims to detect fatigue for one of the most active skeletal muscles at the elbow joint, which is achievable according to our findings. Thus, we advise athletes to take fatigue into consideration to avoid fatigue-induced injuries. The results presented in this work are useful and represent a solid start for moving into real-world applications for detecting the fatigue level in bicep muscles using wearable sensors.

## Figures and Tables

**Table 1:** Borg G.A. Psychophysical bases of perceived exertion [43].

Perceived Exertion	Borg Rating	Examples
None	6	Reading a book, watching television
Very, very light	7 to 8	Tying shoes
Very light	9 to 10	Chores like folding clothes that seem to take little effort
Fairly light	11 to 12	Walking through the grocery store (without speeding up your breathing)
Somewhat hard	13 to 14	Brisk walking (moderate effort and speeding up your breathing)
Hard	15 to 16	Bicycling, swimming, (vigorous effort and get the heart pounding)
Very hard	17 to 18	The highest level of activity you can sustain
Very, very hard	19 to 20	A finishing kick in a race or activity that you cannot maintain for long

**Table 2:** The increase in the percentage of the time to complete a set, compared to the 1st set.

Axis-Sensor	2nd Set	3rd Set	4th Set	5th Set	Avg.
X-Gyroscope	+2.0%	+6.0%	+17.0%	+33.0%	+14.5%
Z-Gyroscope	+1.7%	+11.0%	+15.0%	+45.0%	+18.2%
Y-Accelerometer	+1.5%	+7.4%	+11.0%	+15.0%	+8.7%
ine Avg./set	+1.7%	+8.1%	+14.3%	+31.0%	

**Table 3:** The percentage of muscular endurance changes represented in vertical shrinks.

Axis-Sensor	2nd Set	3rd Set	4th Set	5th Set	Avg.
X-Gyroscope	+0.7%	+1.2%	−6.3%	−5.2%	−2.4%
Z-Gyroscope	+0.5%	+1.7%	−10.4%	−7.5%	−3.9%
Y-Accelerometer	+0.6%	+0.3%	+0.3%	+0.4%	0.4%
ine Avg./set	+0.6%	+1.1%	−5.5%	−4.1%	

**Table 4:** Fatigue detection confusion matrix.

		Actual	
		Fatigue ∈ [17,20]	Non-Fatigue ∈ [6,16]
**Predict**	**Fatigue ∈ [17,20]**	TRUE Fatigue	FALSE Fatigue
	**Non-Fatigue ∈ [6,16]**	FALSE Non-Fatigue	TRUE Non-Fatigue

**Table 5:** Table of the significant (✓) and insignificant (×) features extracted from both fatigue subset and complete dataset; the overlapping features are in highlighted bold.

			Fatigue Subset			Complete Dataset		
			Mean	SD	AAD	Mean	SD	AAD
Sensors and Axes	Gyro.	X-axis	×	×	×	×	×	×
		Y-axis	✓	×	✓	✓	✓	×
		Z-axis	✓	✓	✓	✓	✓	×
	**Mag.**	X-axis	×	×	×	×	×	×
		Y-axis	×	✓	×	×	×	×
		Z-axis	×	×	✓	×	✓	✓
	**Acc.**	X-axis	✓	✓	✓	×	×	×
		Y-axis	✓	✓	✓	✓	✓	✓
		Z-axis	✓	✓	✓	✓	✓	✓
		Total	✓	✓	✓	✓	✓	✓
		Ex. Force	✓	✓	✓	✓	✓	✓

**Table 6:** Average precision, recall, and accuracy for subject-specific validations using the 16 extracted features to detect fatigue in biceps repetitions.

Models	Subject-Specific			
	Precision	Recall	Accuracy	F1
GLM	86%	83%	84%	84%
LR	81%	77%	79%	79%
RF	78%	76%	76%	77%
DT	66%	61%	58%	63%
FNN	95%	93%	94%	94%

**Table 7:** Average precision, recall, and accuracy for cross-subject validations using the 16 extracted features to detect fatigue in biceps repetitions.

Models	Cross-Subject			
	Precision	Recall	Accuracy	F1
GLM	78%	71%	75%	74%
LR	73%	74%	76%	73%
RF	69%	73%	70%	71%
DT	47%	49%	43%	48%
FNN	87%	89%	88%	88%

## Data Availability

The data used in the study is made publicly available at—https://zenodo.org/record/3698242#.XmFZ5qhKguU.
